# The sound of tumor cell-microenvironment communication – composed by the Cancer Cluster Salzburg research network

**DOI:** 10.1186/s12964-017-0176-z

**Published:** 2017-06-02

**Authors:** Silja Wessler, Fritz Aberger, Tanja N. Hartmann

**Affiliations:** 1 Cancer Cluster Salzburg, Department of Molecular Biology, Paris-Lodron University of Salzburg, A-5020 Salzburg, Austria; 2 Cancer Cluster Salzburg, Salzburg Cancer Research Institute (SCRI) - Laboratory for Immunological and Molecular Cancer Research (LIMCR), A-5020 Salzburg, Austria

According to the “hallmarks of cancer” model proposed by Hanahan and Weinberg [1], malignant development results from sustained proliferative signaling, evading growth suppressors, resisting cell death, enabling replicative immortality, inducing angiogenesis, metabolic rewiring, activating invasion and metastasis, and the oncogenic modulation of the anti-tumoral response. Reflecting this highly complex multistep process, tumor initiation and progression are directly caused by genetic mutations and epigenetically modified gene expressions, which drive normal cells towards uncontrolled cell proliferation and survival [1]. During the last decades, it has become clear that tumor development originated by reprogrammed epithelial, mesenchymal, bone marrow or blood cells also depends on a specific tumor environment in which non-cancerous and cancer cells are embedded [2, 3]. In this context, multiple reciprocal communication processes between cancer and stromal cells such as cancer-associated fibroblasts (CAFs), tumor-associated macrophages (TAMs), and additional cells from the immune system are involved in the progression of malignant development [3]. In this review series, researchers from the Cancer Cluster Salzburg (CCS) provide a collection of articles about selected cancer entities, technologies and oncogenic signaling pathways in the context of tumor-microenvironment communication and promising therapeutic approaches.

Inhibitors of DNA binding and cell differentiation (Id) proteins are considered as key players in the regulation of cell cycle progression and cell differentiation [4, 5]. The current knowledge about Id proteins and how they modulate cell cycle regulators is summarized by Roschger and Cabrele [6]. The molecular function of Id proteins is mainly mediated by specific protein-protein interaction via different motifs recognizing structurally diverse proteins. Based on this, binding of Id proteins to class I and II bHLH transcription factors, retinoblastoma protein or the S5a subunit of the 26 S proteasome may explain why Id protein expression can be correlated with a variety of cellular processes ranging from tumor growth, vascularization, invasiveness, metastasis, chemoresistance to stemness [6].

In recent years, hypomethylating agents were implemented in medical treatments of myelodysplastic syndrome (MDS), chronic myelomonocytic leukemia (CMML) and acute myeloid leukemia (AML) indicating that reactivation of epigenetically silenced genes beneficially contributes to cancer therapies. However, new molecular mechanisms of hypomethylating agents have been proposed including the induction of interferon signaling or an involvement in antigen presentation and inflammation. In the review of Wolff and colleagues [7], the authors discuss the observation that hypomethylating agents also induce the expression of inhibitory immune checkpoint receptors and stimuli, which can facilitate tumor immune evasion, but point to new strategies in tumor resensitization for immune checkpoint inhibitor therapies [7].

Another aspect in cancer treatment has been reviewed by Gruber et al. [8]. Targeted therapies with drugs interfering with signaling molecules involved in cancer cell growth, survival and tumor-microenvironment interactions is a highly attractive option. Therefore, the knowledge about phosphorylation dynamics and kinase substrates is essential for the development of inhibitory compounds specifically targeting signaling molecules. Phosphoproteomic analyses using high-performance liquid chromatography-mass spectrometry contribute to the understanding of molecular cellular programs through the identification of deregulated signaling pathways, and have the potential to unravel the intricate signaling networks of tumor-initiating cancer stem cells (CSC) within their niches [8].

The intercellular communication between tumor cells and the immune system via a unique microenvironment plays a crucially important role in proliferation, survival, invasion and metastasis of tumor cells. T cell exhaustion describes an important mechanism to reduce effector T cell functions as part of the immune escape of cancer cells which has been reviewed by Catakovic et al. [9] since revitalizing exhausted T cells represent a powerful strategy to combat cancer.

How the immune system affects cancer development and progression has also been described by Greil and colleagues [10]. Cancer immunoediting involves alterations of the cellular composition and inflammatory cytokine profile in the tumor environment and provides an important link between cancer cells and the immune system. Importantly cancer-associated mutations could result in the exposure of a different epitope pattern on the surface of tumor cells changing the interference with the immune system. This allows new options in the treatment of cancer as summarized in this review [10].

In cancer cells and within the tumor microenvironment, the Hedgehog (HH)/Glioma-associated oncogene homolog (GLI) signaling pathway plays an important role. The current knowledge of HH/GLI signaling in AML has been summarized in the review by Aberger et al. [11]. AML is highly aggressive and only limited possibilities in treatments are available. Targeting HH/GLI in combination with other emerging therapies might represent a novel strategy in this context. The HH/GLI pathway is also implicated in the development of gastric cancer in patients infected with the human pathogen and class-I carcinogen *Helicobacter pylori* (*H. pylori*). The knowledge about the tumor environment of *H. pylori*-infected patients is little. The review of Wessler et al. describes the complex network of *H. pylori*-controlled micromilieu involving different cells of the gastric epithelium [12].

The current insight into the interplay between tumor cells and the environment is steadily increasing. This special issue provides individual molecular details of these complex interactions, including oncogenic driver signals within the complex tumor microenvironment pointing to new innovative and improved therapeutic strategies combating relapse, metastasis and drug resistance to enhance the patient’s survival.

The Cancer Cluster Salzburg (CCS) has been founded in 2014 by Dr. Richard Greil (Salzburg Cancer Research Institute/University Hospital of the Paracelsus Medical University Clinics Salzburg), Dr. Tanja N. Hartmann (Salzburg Cancer Research Institute/Paracelsus Medical University), and Dr. Fritz Aberger (Paris-Lodron University of Salzburg). CCS joins 16 interdisciplinary research groups from the SCRI/SALK and PLUS and constitutes a smart specialization center for tumor-microenvironment communication. Its mission is to explore novel treatment options targeting tumor-microenvironment/immune system interactions for the patients’ benefit.

## References

[1] Hanahan D, Weinberg RA (2011). Hallmarks of cancer: the next generation. Cell.

[2] Junttila MR, de Sauvage FJ (2013). Influence of tumour micro-environment heterogeneity on therapeutic response. Nature.

[3] Quail DF, Joyce JA (2013). Microenvironmental regulation of tumor progression and metastasis. Nat Med.

[4] Prabhu S, Ignatova A, Park ST, Sun XH (1997). Regulation of the expression of cyclin-dependent kinase inhibitor p21 by E2A and Id proteins. Mol Cell Biol.

[5] Melnikova IN, Christy BA (1996). Muscle cell differentiation is inhibited by the helix-loop-helix protein Id3. Cell Growth Differ.

[6] Roschger C, Cabrele C (2017). The Id-protein family in developmental and cancer-associated pathways. Cell Commun Signal.

[7] Wolff F, Leisch M, Greil R, Risch A, Pleyer L (2017). The double-edged sword of (re)expression of genes by hypomethylating agents: from viral mimicry to exploitation as priming agents for targeted immune checkpoint modulation. Cell Commun Signal.

[8] Gruber W, Scheidt T, Aberger F, Huber CG (2017). Understanding cell signaling in cancer stem cells for targeted therapy - can phosphoproteomics help to reveal the secrets?. Cell Commun Signal.

[9] Catakovic K, Klieser E, Neureiter D, Geisberger R (2017). T cell exhaustion: from pathophysiological basics to tumor immunotherapy. Cell Commun Signal.

[10] Greil R, Hutterer E, Hartmann TN, Pleyer L (2017). Reactivation of dormant anti-tumor immunity - a clinical perspective of therapeutic immune checkpoint modulation. Cell Commun Signal.

[11] Aberger F, Hutterer E, Sternberg C, Del Burgo PJ, Hartmann TN (2017). Acute myeloid leukemia - strategies and challenges for targeting oncogenic Hedgehog/GLI signaling. Cell Commun Signal.

[12] Wessler S, Krisch LM, Elmer DP, Aberger F (2017). From inflammation to gastric cancer – the importance of Hedgehog/GLI signaling in helicobacter pylori-induced chronic inflammatory and neoplastic diseases. Cell Commun Signal.

